# Novel sequences, structural variations and gene presence variations of Asian cultivated rice

**DOI:** 10.1038/sdata.2018.79

**Published:** 2018-05-02

**Authors:** Zhiqiang Hu, Wensheng Wang, Zhichao Wu, Chen Sun, Min Li, Jinyuan Lu, Binying Fu, Jianxin Shi, Jianlong Xu, Jue Ruan, Chaochun Wei, Zhikang Li

**Affiliations:** 1 Institute of Crop Sciences, Chinese Academy of Agricultural Sciences, Beijing 100081, China; 2 School of Life Sciences and Biotechnology, Shanghai Jiao Tong University, Shanghai 200240, China; 3 Agricultural Genomics Institute, Chinese Academy of Agricultural Sciences, Shenzhen 518120, China; 4 Shenzhen Institute for Innovative Breeding, Chinese Academy of Agricultural Sciences, Shenzhen 518120, China; 5 Anhui Agricultural University, Hefei 230036, China

**Keywords:** DNA sequencing, Natural variation in plants, Plant genetics, Genetic variation

## Abstract

Genomic diversity within a species genome is the genetic basis of its phenotypic diversity essential for its adaptation to environments. The big picture of the total genetic diversity within Asian cultivated rice has been uncovered since the sequencing of 3,000 rice genomes, including the SNP data publicly available in the SNP-Seek database. Here we report other aspects of the genetic diversity, including rice sequences assembled from over 3,000 accessions but absent in the Nipponbare reference genome, structural variations (SVs) and gene presence/absence variations (PAVs) in 453 accessions with sequencing depth over 20x. Using either SVs or gene PAVs, we were able to reconstruct the population structure of *O. sativa*, which was consistent with previous result based on SNPs. Moreover, we demonstrated the usefulness of the new data sets by successfully detecting the strong association of the “Green Revolution gene”, *sd1*, with plant height. Our data provide a more comprehensive view of the genetic diversity within rice, as well as additional genomic resources for research in rice breeding and plant biology.

## Background & Summary

With the continued reduction of croplands, we are facing a great challenge of feeding the fast growing world population. Asian cultivated rice (*Oryza sativa* L.) is grown worldwide and is the staple food for half the world population. The rich resources of the global rice germplasms are expected to provide a sustainable solution in future rice improvement. However, the total genomic diversity within the primary gene pool of *O. sativa* remains poorly explored. In order to reveal the comprehensive genomic diversity, ‘The 3,000 rice genomes project (3k RG)’^1,2^ was initiated, in which 3,024 accessions representing the most diversity of the cultivated rice population were selected from 89 countries. The 3,024 rice genomes were sequenced using HiSeq 2000 platform with 500 bp DNA fragment libraries and a total of 17 trillion bases were generated. The sequencing data of the 3,024 rice accessions were freely available at GigaDB^1^ (Data Citation 1), NCBI SRA (Data Citation 2), Amazon cloud (https://aws.amazon.com/cn/public-data-sets/3000-rice-genome/) and Aliyun cloud (http://ricecloud.org/). We have previously reported the single nucleotide polymorphisms (SNP) data derived from these sequencing data^3^, which is very useful for QTL mapping by genome-wide association studies.

Structural variations (SVs) and gene presence/absence variations (PAVs) represent additional dimensions of the total genetic diversity within a species and remain largely unknown in almost all eukaryotes. In this descriptor, we reported the SV data and gene PAV data of *O. sativa*, together with the novel sequences absent in the widely used Nipponbare reference genome IRGSP-1.0, as key results of the in-depth analyses of the sequencing data of the 3k RG^4^.

In this data descriptor, we present the rice SV data obtained by calling against the Nipponbare reference genome using novoBreak^5^ because it had the lowest false positive rate when compared with results from several tools such as BreakDancer^6^ and Delly^7^. This SV data set contains a total number of 93,683 SVs (deletions, inversions and duplications of >100 bps and <1 Mbps, and translocations), or an average of 12,178 SVs per genome detected in the 453 high-quality genomes with sequencing depths ≥20× and mapping depths ≥15×. We also present the PAV data sets of 48,098 full-length protein-coding genes (35,633 Nipponbare reference genes and 12,465 novel genes) and 23,876 gene families in the 453 rice accessions, which were obtained from a “map-to-pan” pipeline^8^. These data not only provide a more comprehensive understanding of the genomic diversity within *O. sativa*, but also provide additional genomic markers for genome-wide association studies of rice.

## Methods

**Figure 1:**
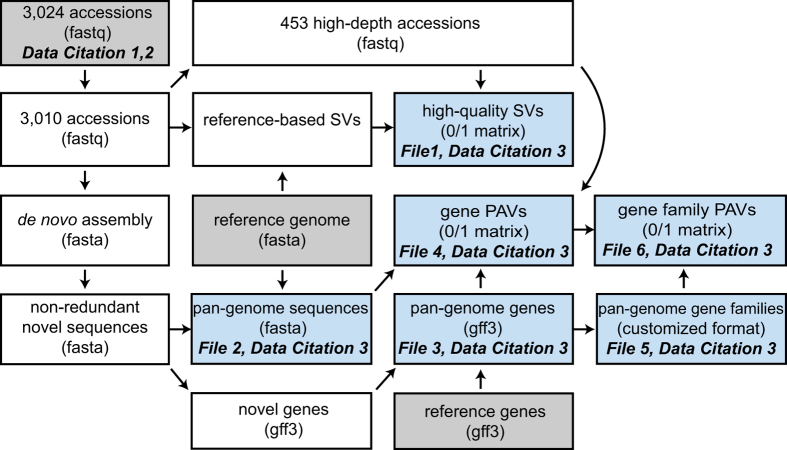
A diagram of data generation and data dependencies. Raw inputs are displayed in grey boxes and important outputs are shown in blue boxes. Data formats are shown in the brackets. The analysis procedure includes: 1) removal of 14 accessions; 2) selection of 453 high-depth accessions; 3) SV calling for 3,010 accessions against the Nipponbare reference genome and extraction of high-quality SVs for the 453 accessions; 4) construction of the pan-genome sequences by combining the Nipponbare reference genome and non-redundant novel sequences; and 5) determination of presence/absence variation of genes and gene families in the 453 accessions with the “map-to-pan” strategy. Detailed methods are described in the Methods section.

In this descriptor, we provided the key outputs of the in-depth analyses in the 3k RG (Fig. 1), including (1) SVs in the 453 high sequencing-depth accessions (File 1, Data Citation 3); (2) the pan-genome sequences of *O. sativa* (File 2, Data Citation 3) and its protein-coding gene annotation (File 3, Data Citation 3); (3) a matrix of gene PAVs of the 453 accessions (File 4, Data Citation 3); and (4) gene family annotation (File 5, Data Citation 3) and a matrix of gene family PAVs of the 453 accessions (File 6, Data Citation 3). The methods described below are expanded versions of descriptions in our related work^4^.

The sequencing data of 3,024 accessions of 3k RG were available from various sources (Data Citation 1 and Data Citation 2). To begin with, 14 accessions were removed from the 3,024 sequenced accessions. Accessions CX400, CX401, CX402, IRIS_313-11415 and IRIS_313-10729 belong to African cultivated rice (*Oryza glaberrima* L.). Accessions IRIS_313-8502, IRIS_313-9233, IRIS_313-8444, IRIS_313-10057, IRIS_313-9184, B014, IRIS_313-9404 were removed due to significant contamination, and accession B101 was removed due to a very small estimated genome size. Accession IRIS_313-8921 was removed due to its extremely low sequencing depth (0.3×).

The sequencing depths of the remaining 3,010 genomes ranged from 4.2x to 63.8x. Clean reads of each accession were mapped to the Nipponbare reference genome (IRGSP-1.0) using “bwa mem” with default parameters^9^. The mapping depth was calculated as total mapped bases divided by the size of IRGSP-1.0 genome. Only the 453 rice accessions with sequencing depth ≥20 and mapping rate ≥15 were selected for reporting final SVs and PAVs.

### Structural variations

novoBreak^5^(https://sourceforge.net/projects/novobreak/?source=navbar) was used for SV calling against the Nipponbare genome. The comparison of performances of three software, novoBreak^5^, BreakDancer^6^ and Delly^7^ are available in our related work^4^. novoBreak was first run on each of the 3,010 accessions independently with default parameters. We detected deletions, inversions and duplications with sizes between 100 bps and 1Mbps, and translocations (interchromosomal breakpoints). SVs which could be inferred by no less than 3 kmer/reads were further selected with the following filter conditions: 1) more than 4 supporting split reads; or 2) no less than 3 discordant read pairs for both breakpoint. To identify SVs in different samples, all SVs passed the filter condition in the 453 accessions were pooled together to justify coordinated variants. Any two adjacent SVs were identified as a single SV if their start and end positions varied no more than 1 kb, and their overlapping region is over half of the total size. The presence/absence matrix of SVs in each accession was built with these criteria. To minimize the false positive rates, SVs detected in less than 6 accessions or more than 80% of the 3,010 accessions were removed. The SVs in the 453 high-depth accessions were then extracted as a more confident subset (File 1, Data Citation 3).

### Novel sequences

Novel sequences absent in the reference genome were derived from the *de novo* assemblies of the 3,010 rice accessions. To achieve high-quality assemblies, we utilized a method with iterative use of SOAPdenovo r240^10^ to select the best Kmer parameter for each accession. In detail, we first determined an initial Kmer (K_*init*_) for rice accession *R* based on a linear model K_*init*_=ROUND(a*Dep_*R*_+*b*), where Dep_*R*_ is the sequencing depth of *R*, and *a* and *b* are the parameters of the linear model. This linear model was trained on 50 randomly selected rice accessions and the initial Kmer was set as ‘K=2*int (0.38*(sequencing depth) +10)+1’ (R2=0.68). After K_*init*_ was determined, we ran SOAPdenovo 3 times with stepped Kmers (Kmer_*low*_=Kmer_*init*_- 2, Kmer_*mid*_=Kmer_*init*_ and Kmer_*high*_=Kmer_*init*_+2) using command line “SOAPdenovo-63mer(or SOAPdenovo-127mer) all -s configure_file (average insertion length set as 460 in the configure file) -o output_directory -K Kmer -R -F -p 8”; Gapcloser tool within SOAPdenovo package was then used on the SOAPdenovo raw scaffolds with command “GapCloser -b configure_file (the same as the configure file used in running SOAPdenovo) -a soap_raw.scaf -o output_gc.scaf -t 8”; Gap closed scaffolds were then broken down to contigs by cutting scaffolds at sites with >10 consecutive Ns. The *N50* values of the gap-closed contigs were calculated for Kmer_*low*_, Kmer_*mid*_ and Kmer_*high*_ respectively. If N50 of Kmer_*mid*_ was the highest, assembly at Kmer_*mid*_ was selected as the final result. If N50 of Kmer_*high*_ was the highest, an additional run with Kmer=Kmer_*high*_+2 was needed and we compared N50 of Kmer_*mid*_, Kmer_*high*_ and Kmer_*high*_+2; this iteration continued until *N50* of the median Kmer became the highest. If *N50* of Kmer_*low*_ was the highest, Kmer went down and similar iteration was carried out to determine the best Kmer. In order to control the false positives, though GapCloser was involved in the assembly pipeline and provided larger N50, only raw contig assemblies from SOAPdenovo were used for detection of novel sequences.

Assemblies were assessed by comparing to the Nipponbare reference genome using QUAST v2.3^11^, and those unaligned contigs were then selected. All the unaligned contigs with length >500 bp from each rice accession were merged into a single sequence set. Next, CD-HIT v4.6.123^12^ was used to remove redundant sequences at an identity cut-off of 90% with command “cd-hit-est -i input.fa -o output.fa -c 0.9 -T 16 -M 50000”. This process was carried out for 3 times to ensure redundancies were removed. Next, various contaminants including archaea, bacteria, virus, fungi and metazoa were removed. In practice, the non-redundant sequences were aligned to the NT database (07/26/2014) with NCBI-blast v2.2.28+ with command “blastn -db nt.fa -task megablast -query input.fa -num_threads 16 -evalue 1e-5 -out o -outfmt \"6 qseqid sseqid qlen length qstart qend sstart send pident evalue\” -best_hit_overhang 0.25 -perc_identity 0.5 -max_target_seqs 10”. Contigs whose best alignments (E-value as 1^st^ keyword and identity as 2^nd^ keyword) were not in Viridiplantae (annotation information downloaded from NCBI taxonomy database) were considered as contaminants and were filtered out. The remaining contigs formed the non-redundant novel sequence dataset (identity<0.9 in comparison to IRGSP genome and identity<0.9 among the novel sequences themselves). In order to be browsed in a genome browser, the novel contigs were concatenated with 100 consecutive Ns as the delimiters. The reference sequences and the novel sequences were combined to form the rice pan-genome sequences (File 2, Data Citation 3).

### Gene presence/absence variations

Both the Nipponbare reference genes and non-Nipponbare novel genes were included in the PAV analyses. The gene annotation of IRGSP-1.0 genome was downloaded from the Rice Annotation Project (RAP)^13^. In order to be consistent with gene annotation for non-IRGSP sequences, also to simplify the subsequent analyses, only those transcripts with the longest open reading frame (ORF) were selected as representatives. Protein-coding genes on the novel sequences were predicted using MAKER2^14^ (a gene prediction pipeline combining *ab initio* predictions, expression evidence and protein homologies). In detail, low-complexity repeats were first masked. Two *ab initio* predictors, snap^15^ and AUGUSTUS^16^, were utilized to predict gene models with their default parameters for rice. All rice ESTs were downloaded from Genbank (12/15/2014) and were aligned to the novel sequences with NCBI-blastn. All rice proteins were downloaded from NCBI (12/15/2014) and were aligned to the novel sequences with NCBI-blastx. To get more informative alignments, Exonerate^17^ was used to realign each sequence identified by blast around splice sites. EVidenceModeler^18^ was used to combine and refine the *ab initio* predictions with RNA and protein evidences. Incomplete gene models were further removed. Since we used a method based on representative sequences to build the novel sequences, there might be local similarities between novel sequences and the reference genome, as well as among novel sequences themselves. Therefore, there might also be genes predicted in the novel sequences similar to genes in the reference genome and also novel genes similar to each other. In order to remove these genes, we clustered all genes at global protein identity of 95%, and remove those novel genes who are not the representative of the group. The non-redundant novel genes together with the reference genes formed the rice pan-genome (File 3, Data Citation 3).

We utilized a “map-to-pan” strategy^8^ to determine gene presence/absence based on the observation that more than 98% of the genome can be covered by short read mapping at sequencing depth ≥20. The presence/absence of each gene was determined by the read coverage of the gene body and its coding region. In practice, we first mapped reads of each accession to the pan-genome sequences with “bwa mem” and a gene was considered as present if it had 1) coding coverage >0.95 (covered bases in the ORF divided by the ORF length) and 2) gene body coverage >0.85 (the number of covered bases in the gene body divided by the gene length). The presence/absence information of each gene from the 453 high sequencing-depth accessions were then merged together as a 0/1 matrix (File 4, Data Citation 3).

### Gene family presence/absence variations

Similar to a recent pan-genome study on soybean^19^, the genes were clustered to gene families with OrthoMCL v2.0.9^20^ at protein similarity cut-off of 0.5. In detail, all coding sequences of genes on the pan-genome sequences were first extracted and then translated into protein sequences. All protein sequences were compared by using all-by-all blastp (Evalue set as 1e-5). OrthoMCL was used to process the blastp output and cluster genes to gene families (File 5, Data Citation 3).

Gene family presence/absence (File 6, Data Citation 3) was further determined with a straightforward rule based on gene presence/absence. If at least one gene of a gene family presented in the rice accession, this gene family was considered to be present in this rice accession.

### Code availability

All codes for detection of the novel sequences and determination of gene PAVs were implemented in EUPAN package^8^ available at http://cgm.sjtu.edu.cn/eupan/. Software and their used versions were described as above.

## Data Records

The raw data of ‘The 3,000 rice genome project’ (3k RG) were deposited in GigaDB (Data Citation 1) and NCBI’S Short Read Archive (SRA) under accession number PRJEB6180 (Data Citation 2). Data records presented in this descriptor are available online from Figshare. The presence/absence matrix of SVs including deletion, duplication, inversion and translocations (File 1, Data Citation 3) contains both SV annotations and their presence/absence in 453 high-depth rice accessions. The pan-genome sequences (File 2, Data Citation 3) include both the IRGSP-1.0 sequences and novel sequences. The protein-coding gene annotation (File 3, Data Citation 3) is in GFF3 format. The gene family annotation file (File 5, Data Citation 3) is formatted as a gene family id followed by all its gene members in a line. The gene PAV matrix file (File 4, Data Citation 3) and gene family PAV matrix file (File 6, Data Citation 3) are formatted as a matrix with values of either 0 (absence) or 1 (presence). Each line in the matrix represents a gene/gene family and each column represents a rice accession.

## Technical Validation

**Figure 2:**
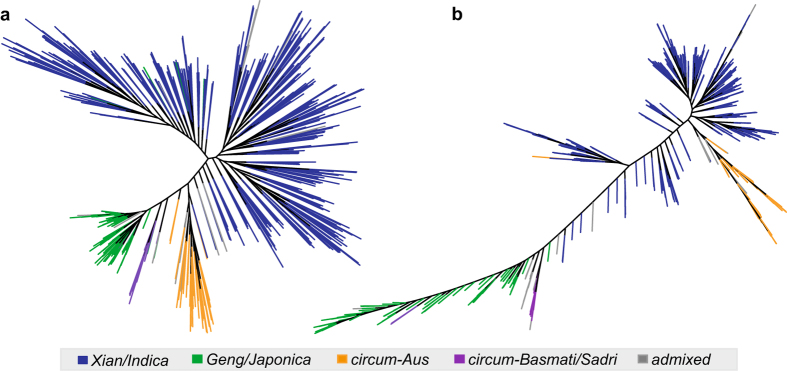
Phylogenetic analyses of the 453 rice accessions based on SVs and gene PAVs. (**a**) the phylogenetic tree constructed from SVs of the 453 accessions; (**b**) the phylogenetic tree constructed from gene PAVs of the 453 accessions. Different Colors represent the grouping information derived from SNP analyses. Both SVs and PAVs clearly classified the 453 rice accessions into *Xian/Indica*, *Geng/Japonica*, *circum-Aus* and *circum-Basmati/Sadri* groups.

Based on the SNP data, the 3,010 rice accessions were clearly divided into *Xian*-*Indica*, *Geng*-*Japonica*, circum-Aus and circum-Basmati groups, together with some admixed accessions^4^. Here we reconstructed the phylogenetic relationship of the 453 rice accessions based on SVs and gene PAVs with “pars” tool of the phylip package (http://evolution.genetics.washington.edu/phylip.html). As a result, both the SVs and the gene PAVs showed consistent phylogenies (Fig. 2a and b), suggesting the validity of these data.

**Figure 3:**
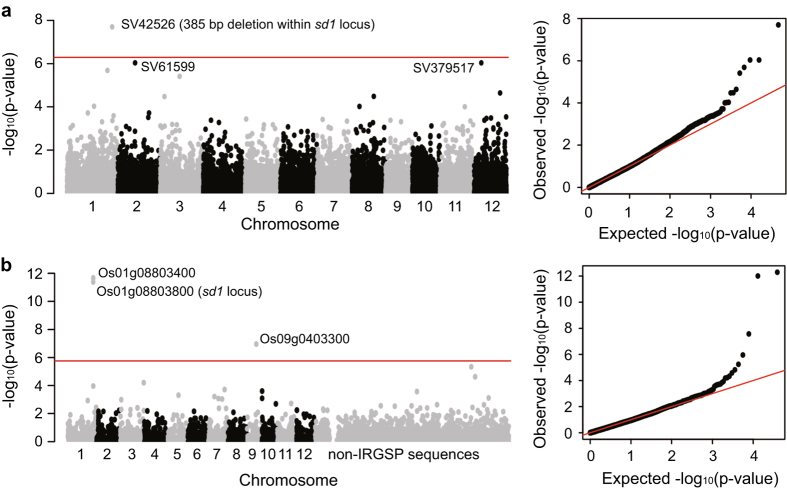
Genome-wide association studies of plant height based on both SVs and gene PAVs. (**a**) genome-wide association study based on SVs including deletions, duplications and inversions; (**b**) genome-wide association study based on PAVs of distributed genes. The left panels show the Manhattan plots and the right panels show the quantile-quantile plots. The thresholds of significant *P*-values were 5.1e-7 and 4.2e-7, respectively (with bonferroni correction, significant level at 0.01).

In order to demonstrate the usefulness of these data, we carried out genome-wide association studies of plant height, an agronomically important phenotype, based on both the SVs and the gene PAVs. For 323 out of the 453 rice accessions, the plant height data were collected. SVs including deletions, duplications and inversions were utilized for the analysis. Efficient mixed model analyses (EMMA) by EMMAX software^21^ were used for the association studies. In detail, BN kinship matrixes were calculated to measure the genetic similarity between accessions based on the genome-wide SV data and gene PAV data, respectively. As a result, GWAS based on both SVs (Fig. 3a) and gene PAVs (Fig. 3b) successfully detected the *sd1* locus, the well-known “Green Revolution gene”, whose loss of function is associated with the greatly reduced plant heights^22^. Moreover, we also detected several other candidate variations significantly associated with plant heights.

This paper released two important types of variations beyond SNPs for rice. This is also the first release of gene presence/absence variations among hundreds of individuals for a higher eukaryote. We demonstrated the potential of these variations to explain phenotype variations.

## Additional information

**How to cite this article**: Hu Z. *et al.* Novel sequences, structural variations and gene presence variations of Asian cultivated rice. *Sci. Data* 5:180079 doi: 10.1038/sdata.2018.79 (2018).

**Publisher’s note**: Springer Nature remains neutral with regard to jurisdictional claims in published maps and institutional affiliations.

## Supplementary Material
